# Remote Recurrent Primary Hyperparathyroidism in Auto-Transplanted Tissue

**DOI:** 10.7759/cureus.40715

**Published:** 2023-06-21

**Authors:** Melisa Esposito, Thomas Koroscil

**Affiliations:** 1 Internal Medicine, Kettering Health Network, Dayton, USA; 2 Endocrinology, Wright State University Boonshoft School of Medicine, Dayton, USA

**Keywords:** parathyroidectomy, osteopenia, pheochromocytoma, medullary thyroid carcinoma, parathyroid hyperplasia, hypercalcemia, recurrent primary hyperparathyroidism, auto-transplantation, men2a, primary hyperparathyroidism

## Abstract

Multiple endocrine neoplasia type 2A (MEN2A) is a rare hereditary condition characterized by medullary thyroid cancer, pheochromocytoma, and primary hyperparathyroidism. The current standard of treatment of hyperparathyroidism involves surgical removal of visibly enlarged glands, and auto-transplantation of remnant tissue is often considered to minimize the risk of iatrogenic post-surgical hypocalcemia if multiple glands are enlarged. Rarely, hyperparathyroidism may recur due to hyperplasia or adenoma formation in the auto-transplanted tissue. The following case portrays a 51-year-old male, with a history of MEN2A status post total parathyroidectomy with cryopreservation and subsequent auto-transplantation of remnant parathyroid tissue to the left arm 18 years prior, who presented to establish care due to the insidious development of asymptomatic hypercalcemia. Workup included a laboratory examination showing elevated intact parathyroid hormone (PTH) and left arm ultrasound revealing three areas of enlarged parathyroid tissue at the transplant site, raising suspicion for the development of recurrent primary hyperparathyroidism in auto-transplanted tissue. The patient ultimately underwent a re-do subtotal parathyroidectomy of auto-transplanted tissue with surgical pathology confirming hyperplastic parathyroid tissue. This case highlights the significance of indefinite vigilant surveillance in this patient population, as a recurrence of hyperparathyroidism may occur even after decades of remission.

## Introduction

Multiple endocrine neoplasia type 2 (MEN2) is a rare hereditary condition characterized by medullary thyroid cancer, pheochromocytoma, and primary hyperparathyroidism [1]. The management of hyperparathyroidism typically requires total or subtotal parathyroidectomy, and auto-transplantation of the remnant tissue to the forearm or sternocleidomastoid muscle is commonly pursued for the prophylaxis or treatment of iatrogenic post-surgical hypoparathyroidism [2]. Rarely, primary hyperparathyroidism may recur due to hyperplasia or adenoma formation in the auto-transplanted tissue [2-4]. Here, we present a rare case of recurrent primary hyperparathyroidism presenting 18 years after parathyroidectomy.

## Case presentation

**Figure 1:**
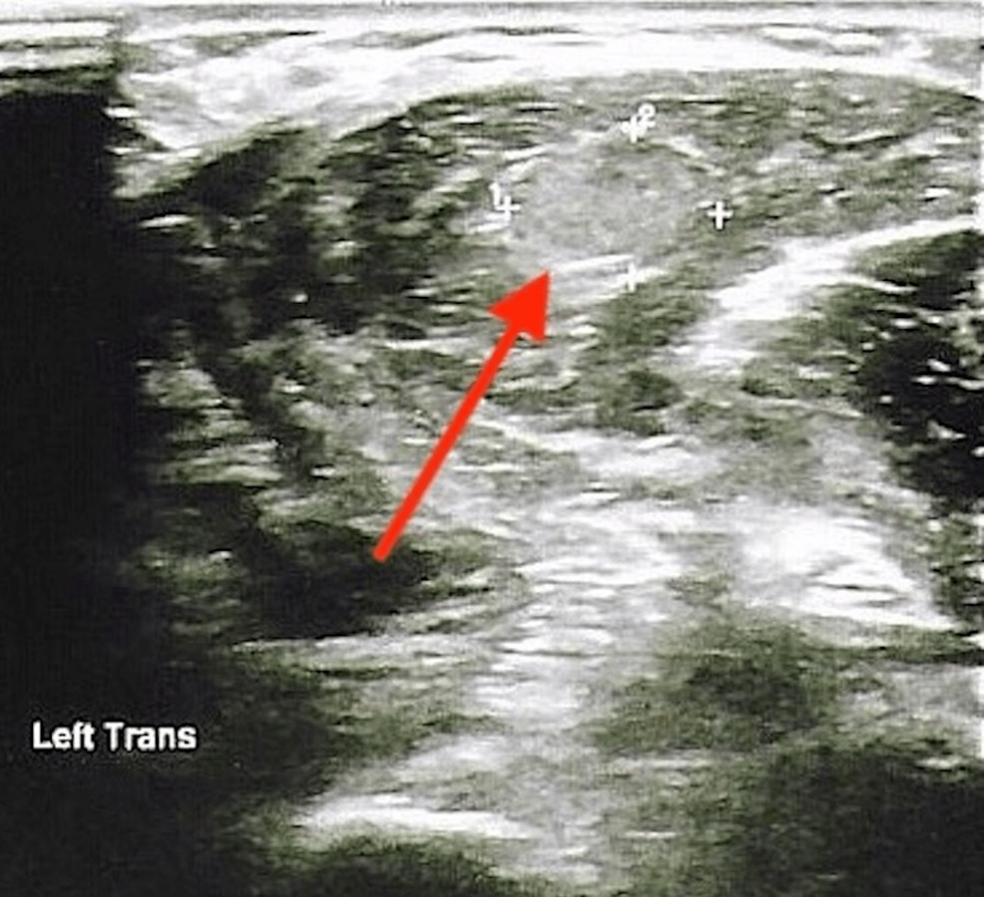
Left forearm ultrasound transverse view revealing an auto-transplanted parathyroid adenoma (red arrow)

**Figure 2:**
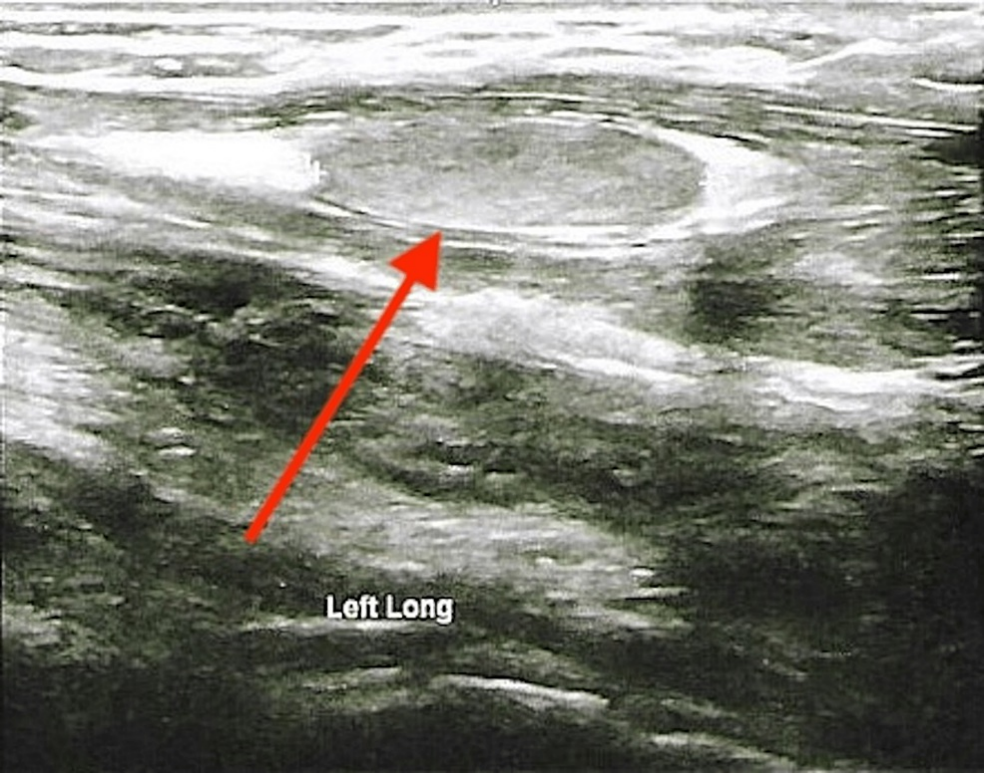
Left forearm ultrasound longitudinal view revealing an auto-transplanted parathyroid adenoma (red arrow)

A 51-year-old male with multiple endocrine neoplasia type 2A (MEN2A) presented with concerns for new-onset hypocalcemia. Relevant medical and surgical history included medullary thyroid cancer status post total thyroidectomy (on levothyroxine), bilateral pheochromocytomas status post bilateral adrenalectomy (on prednisone and fludrocortisone), and primary hyperparathyroidism status post parathyroidectomy. The patient had undergone total parathyroidectomy with cryopreservation of parathyroid tissue 18 years prior. Following initial surgery, the patient was severely hypocalcemic, with labs revealing serum calcium as low as 6.0 mg/dL (reference range (RR) 8.5-10.5 mg/dL), phosphorous 3.9 mg/dL (RR 2.2-4.5 mg/dL), albumin 3.0 g/dL (RR 3.2-4.4 g/dL), and creatinine 0.93 mg/dL (RR 0.7-1.3 mg/dL). Given no evidence of parathyroid hormone (PTH) production for several months following surgery, the patient ultimately underwent auto-transplantation of cryopreserved tissue into the left forearm. The auto-transplanted parathyroid tissue slowly began to function and the patient's calcium gradually increased to normal limits. The patient underwent regular surveillance labs and calcium levels remained stable for several years following auto-transplantation. On presentation to the office, the patient was noted to have serum calcium of 11.2 mg/dL with intact PTH of 128 pg/mL (RR 18-65pg/mL). 25-hydroxy vitamin D of 32 ng/mL (RR 30-100 ng/mL), and renal function was otherwise within normal limits, suggesting primary hyperparathyroidism. Most recent labs three years prior revealed normal serum calcium of 8.5 mg/dL with PTH of 20 pg/mL. The patient was asymptomatic on presentation and denied kidney stones, difficulty concentrating, abdominal pain, reflux, arthralgias/myalgias, increased urination, or any other concerns. Physical examination revealed a nontender area of fullness over the left forearm. Ultrasound of the left forearm transplant site revealed three discrete isoechoic areas representative of parathyroid tissue, the largest of which measured approximately 10x7.5x20 mm (normal size 3x5x7 mm) (Figures 1, 2). The neck ultrasound was unrevealing. Subsequent dual X-ray absorptiometry (DEXA) scan was consistent with osteopenia, with T-scores of -1.6 in the right femoral neck, -1.5 in the left femoral neck, and -1.1 in bilateral hips. This reflected a 4.4% bone mineral density loss in the left total hip and a 4.5% loss in the right total hip as compared to a previous study two years prior, prompting consultation with endocrine surgery. Further workup included a sestamibi scan, which revealed no evidence of parathyroid tissue in the neck, and differential intact PTH measurement, which yielded a value of 1,771.7 pg/mL in the left arm and 171.8 pg/mL in the right. The patient consequently underwent redo subtotal parathyroidectomy with the removal of the largest mass and half of a second mass, leaving additional residual tissue in place so as to prevent iatrogenic post-surgical hypoparathyroidism. Surgical pathology revealed hyperplastic parathyroid tissue, confirming the diagnosis of recurrent primary hyperparathyroidism in auto-transplanted tissue.

## Discussion

Multiple endocrine neoplasia type 2 (MEN2) is an autosomal dominant disorder that predisposes toward the development of endocrine neoplasia [5]. Prevalence is estimated at one per 30,000 in the general population [6]. MEN2 is subclassified into two distinct syndromes, types 2A (MEN2A) and 2B (MEN2B), both of which are associated with germline gain-of-function mutations in the RET protooncogene on chromosome 10q11.2 [5,7]. First described by Steiner et al. in 1968, MEN2A is characterized by the development of medullary thyroid cancer (MTC), pheochromocytoma, and primary hyperparathyroidism [1]. MTC is the dominant component of the disease, affecting essentially 100% of patients who inherit the RET protooncogene mutation [6]. Development of pheochromocytoma and hyperparathyroidism is far more variable, occurring in 40-50%% and 15-30% of patients, respectively [8]. MEN2A should be suspected in patients exhibiting classical clinical features and family history, though genetic testing with identification of germline RET mutation typically confirms the diagnosis [9].

In terms of treatment, it is important to note that almost 100% of patients with MEN2A will develop MTC [6,9]. As such, therapeutic total thyroidectomy is recommended for all patients with hereditary forms of MTC, and prophylactic thyroidectomy is advised in individuals with known RET mutations [9,10]. Preoperative evaluation prior to thyroidectomy should include testing for coexisting tumors, with measurement of serum calcium to rule out concomitant hyperparathyroidism and plasma fractioned metanephrines as the initial screening test for pheochromocytoma. If pheochromocytoma is detected, it should be removed prior to thyroidectomy or parathyroidectomy [2]. If initial screening tests for coexisting tumors are negative, continued screening for these conditions should continue on a yearly basis [11].

Generally speaking, surgical resection of hyperfunctional parathyroid tissue is the only definitive treatment option for patients with primary hyperparathyroidism [12]. However, determining the optimal surgical management of hyperparathyroidism in patients with MEN2A has historically been challenging given morphologically heterogenous parathyroid glands and the risk of hypoparathyroidism associated with initial or re-operative thyroidectomy for MTC [8]. Several surgical options exist, including resection of only visibly enlarged parathyroid glands, subtotal parathyroidectomy with one gland or a portion of one gland left in situ, or total parathyroidectomy with heterotopic auto-transplantation [8,12-15].

In a study of 119 patients with MEN2A in 1996, Herfarth et al. reported persistent or recurrent primary hyperparathyroidism occurring only after selective (9%) or subtotal (14%) resection, with an equivalent rate of permanent hypoparathyroidism after subtotal vs total parathyroidectomy (29% vs 20%, respectively), providing a rationale for total parathyroidectomy with forearm autograft [12]. However, in a separate study of 56 MEN2A patients performed that same year, Kraimps et al. reported that cure was achieved in only 89% of patients, irrespective of the extent of resection [16]. Eleven percent (11%) had persistent hyperparathyroidism and 9% had recurrent hyperparathyroidism during a follow-up period of 6.4 years [16]. Combined with a rate of hypoparathyroidism in 22% of patients, the authors concluded that resection of only macroscopically enlarged glands was sufficient [16]. Taking these results into consideration, in 2016, the American Association of Endocrine Surgeons released guidelines stating that in patients with primary hyperparathyroidism in the setting of MEN2A who have not had previous neck surgery, resection may be limited to only visibly enlarged glands [8]. If all four glands are abnormal, subtotal parathyroidectomy should be performed and part of one parathyroid gland should be left in situ on a vascular pedicle or auto-transplanted to a heterotopic location so as to preserve parathyroid function [8,10]. The principal advantage of auto-transplantation is that it decreases the frequency of permanent hypoparathyroidism and facilitates the removal of hyperfunctional tissue should recurrence occur [3].

Cure or failure (persistence or recurrence) of primary hyperparathyroidism is usually determined at the six-month time mark, with “cure” typically being defined as normocalcemia lasting a minimum of six months [8]. Inversely, failure is classified as either persistent or recurrent disease, defined as hypercalcemia occurring within the first six months or greater than six months following parathyroidectomy, respectively [8]. Diagnosis of either condition requires the absence of exogenous calcium administration and the exclusion of causes of secondary hyperparathyroidism [8]. Given the strong genetic predisposition for recurrence in patients with conditions associated with familial hyperparathyroidisms, such as MEN2A, achieving a sustained lifelong cure may be challenging. In these cases, the likelihood of recurrence is not a question of “if”, but rather “when” [8]. Therefore, the primary objective of surgery in these patients is not necessarily to achieve a permanent cure but rather to extend the time before recurrence, prevent iatrogenic hypocalcemia and facilitate future surgery in the event of recurrent disease [8,17].

Following surgery, patients should undergo yearly screening for recurrence with an annual measurement of serum calcium [15]. Few cases of recurrent primary hyperparathyroidism secondary to auto-transplanted parathyroid adenoma exist in the literature [3,4]. This case is particularly rare in that it represents the most protracted time course, with 18 years from initial auto-transplantation to the development of clinically detected disease. This clinical scenario highlights the importance of vigilant, continued surveillance of serum calcium in this patient population, particularly in the setting of MEN2A, as recurrence may occur even following decades of remission.

## Conclusions

MEN2A is associated with the development of MTC, pheochromocytoma, and primary hyperparathyroidism. Surgery is the mainstay of treatment in patients who develop primary hyperparathyroidism. While several surgical options exist, subtotal parathyroidectomy with resection of only visibly enlarged glands is recommended. Following surgery, remnant tissue may be left in situ on a vascular pedicle or auto-transplanted to a heterotopic location to reduce the risk of postoperative iatrogenic hypocalcemia. Given the genetic predisposition for hyperparathyroidism in patients with MEN2A, recurrence is often considered inevitable. Therefore, watchful vigilance is essential, and patients should be screened for recurrence with yearly serum calcium measurements following parathyroidectomy. Screening should continue indefinitely, as recurrence can occur even after decades of remission.
